# The Response of Greek Key Proteins to Changes in Connectivity Depends on the Nature of Their Secondary Structure

**DOI:** 10.1016/j.jmb.2015.03.020

**Published:** 2015-06-19

**Authors:** Katherine R. Kemplen, David De Sancho, Jane Clarke

**Affiliations:** University of Cambridge Department of Chemistry, Lensfield Road, Cambridge CB2 1EW, UK

**Keywords:** MD, molecular dynamics, WT, wild type, protein folding, topology, death domain, immunoglobulin fold, phi-value

## Abstract

What governs the balance between connectivity and topology in regulating the mechanism of protein folding? We use circular permutation to vary the order of the helices in the all-α Greek key protein FADD (*F*as-*a*ssociated *d*eath *d*omain) to investigate this question. Unlike all-β Greek key proteins, where changes in the order of secondary structure cause a shift in the folding nucleus, the position of the nucleus in FADD is unchanged, even when permutation reduces the complexity significantly. We suggest that this is because local helical contacts are so dominant that permutation has little effect on the entropic cost of forming the folding nucleus whereas, in all-β Greek key proteins, all interactions in the nucleus are long range. Thus, the type of secondary structure modulates the sensitivity of proteins to changes in connectivity.

## Highlights

• What is the relative importance of connectivity and topology in determining folding mechanisms?
• We use circular permutation change the connectivity of the helices in a Greek key protein, FADD.
• Unlike all-β Greek key proteins, the position of the nucleus in all-α FADD is unchanged.
• Secondary structure content of proteins determines their response to changes in connectivity.

## Graphical abstract

**Figure:**
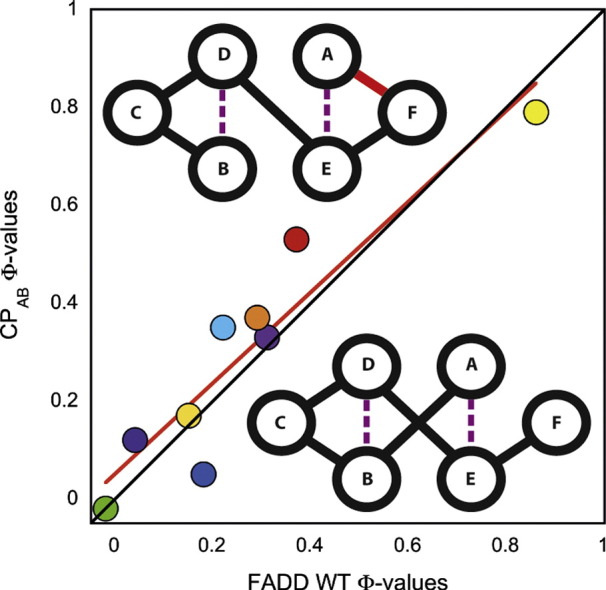

## Introduction

The topology of a protein is an important determinant of its folding mechanism and kinetics [1–5]. We have previously compared the folding mechanism of FADD (*F*as-*a*ssociated *d*eath *d*omain), an all-α Greek key domain formed of two 3-helix bundles, with the folding of spectrin domains (simple 3-helix bundles) and with Greek key all-β immunoglobulin (Ig)-like domains. We found that the helices that form the central core, two from each bundle, align first, with the peripheral helices packing late. We ascribed this to the complexity of the Greek key topology, as we see a similar folding mechanism, involving assembly of elements of structure distant in sequence but central to the structure in Ig-like domains. The difference is that, in the helical FADD domain, secondary structures (i.e., local helical contacts) are obliged to form simultaneously, whereas tertiary contacts dominate in Ig-like folding. In contrast, in the simple 3-helical bundle spectrin domains, folding mechanisms are far more malleable [6].

Circular permutation has been used to investigate the importance of chain connectivity in determining mechanism. A circular permutant retains the same amino acid composition and chain length [7,8] as the wild type (WT) but the order of secondary structure elements is altered. Circular permutation may change the relative sequence separation of key residues in the folding nucleus, and hence, such mutants have been termed “entropy mutants” [3]. Several circular permutant systems have been used to investigate affects on stability [9–14] and activity [15–17], but relatively few studies have considered the effect on the kinetics of protein folding [1,3,5,18–25]. Proteins with an assortment of folds including β-trefoil, SH3, β-sandwich and the PDZ domains have been studied. In some cases, a change in mechanism upon mutation has been observed [1,3,5,20,26,27], but not in others [18,25]. What determines the balance between topology and chain connectivity in deciding folding mechanism? Is the nature of the secondary structure important? These previous studies have largely been on proteins with predominantly, or all, β structure, where the interactions within the protein are primarily long range (i.e., the proteins have large relative contact orders). Here, we investigate the all-α protein FADD using a circular permutation strategy to investigate how altering the connectivity of helices, in particular when we create two contiguous helical bundles, affects the folding.

## Results and Discussion

### Circular permutation has little effect on structure

**Fig. 1:**
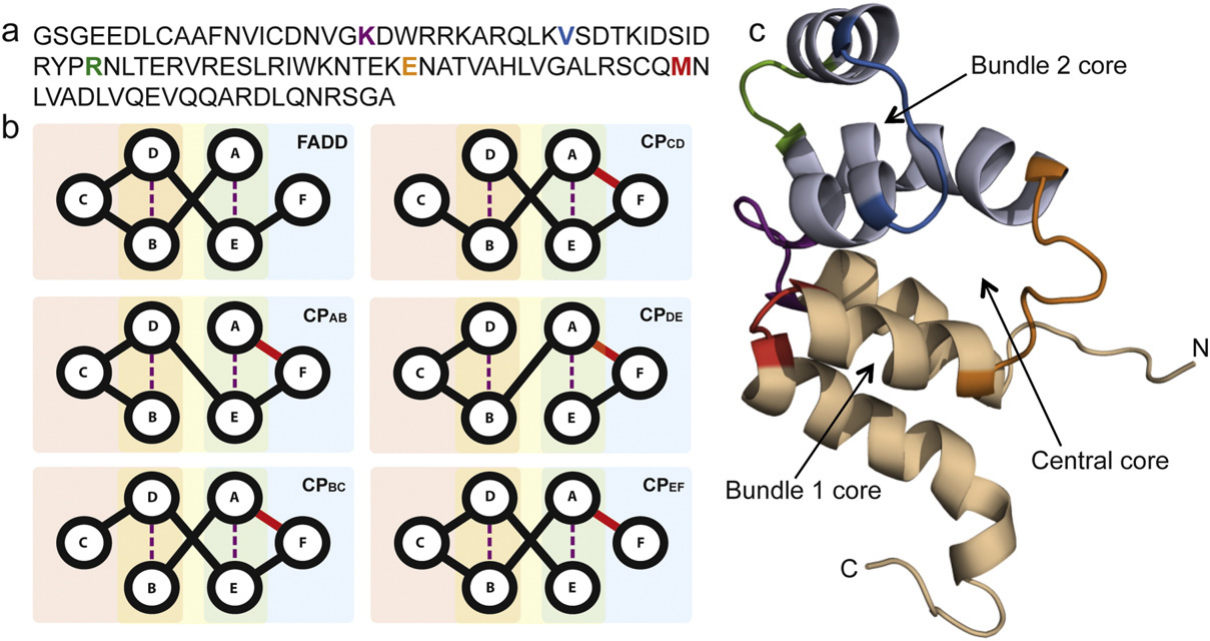
Design of the circular permutants. (a) WT amino acid sequence, with the new N-termini highlighted as follows: purple (CP_AB_), cyan (CP_BC_), green (CP_CD_), orange (CP_DE_) and red (CP_EF_). These colours correspond to the same permutants throughout this paper. (b) Schematics of FADD and circular permutants CP_AB-EF_. Covalent linkage of the WT termini is indicated in red. Bundles of helices are indicated by the background colours blue (B1), yellow (central core) and red (B2) [4]. Helices that pack onto each other within the core are identified by the dotted lines. (c) NMR structure (1E41 [30]) showing B1 in cream and B2 in grey. The loops are coloured to match the corresponding permutant. All permutant genes were synthesised by GenScript, USA. Amino acid sequences included an N-terminal -GSGSS- spacer between the thrombin cleavage site and the protein. The proteins were expressed and purified as described previously [4].

FADD comprises six helices, referred to here as helices A–F, starting from the N-terminus (Fig. 1). The helices are arranged into two 3-helix bundles, each with a hydrophobic core, that pack together to form a central core: bundle 1 (B1) is non-contiguous and is formed by helices A, E and F, whilst bundle 2 (B2) is formed from three contiguous helices B, C and D. The non-contiguous nature of B1 means that the bundles are held in place by two loops (A–B and D–E) that cross either end of the core. In the circular permutants, the native termini have long flexible tails that were joined directly, whilst a flexible 5-residue (-GSGSS-) tail was added to the new N-terminus to ensure that the thrombin cleavage site used during purification was still accessible despite its new proximity to secondary structure elements. The permuted sequences were designed so that the new termini fell within loops connecting the six helices, giving rise to five permutants referred to as CP_AB_, CP_BC_, CP_CD_, CP_DE_ and CP_EF_ (Fig. 1). All five permutants could be successfully expressed and were isolated in a soluble form.

Previous studies of circular permutants have observed little alteration in the native structures [18,19,25,28,29]. We used both experimental probes and molecular dynamics (MD) simulations to assess whether the structure of FADD was significantly altered upon permutation. The extent of secondary structure was determined experimentally using circular dichroism (CD), and all proteins had comparable helical content (Fig. S1a). A consistent loss of helicity compared to WT was observed for all permutants that we ascribe to the increase in flexibility at the sites of the new N- and C-termini, as well as a reduction in helicity at the site of the WT termini due to strain inherent in the new loop. There are two Trp residues in FADD. The Trp fluorescence of all but one of the permutants was similar to WT, suggesting that the hydrophobic cores are similarly packed (Fig. S1b), an observation supported by analysis of simulations of the permuted structures (see below). There was a small shift in λ max for folded CP_DE_, which may reflect slight alterations in the chemical environment of Trp148 that packs on the D-E loop. CP_EF_, however, has a fluorescence profile that is very different to that of the other permutants, with a significant increase in fluorescence in the folded state. This cannot be explained trivially. Loop E-F is not close to either of the Trp residues, and it is likely that there is a change in structure in this permutant; although this is not detected in the simulations, there are other experimental hints (see below).

We ran short atomistic MD simulations of the WT (1E41 [30]) and each of the permutants. The simulations we performed are not long enough to characterise the native-state dynamics comprehensively since these can range from the sub-nanosecond to the microsecond timescale [31]. However, even with their limited length, simulations can indicate the extent of perturbation of the structure upon permutation and how each permutant relaxes to its corresponding ensemble. It is important to be aware that force field deficiencies will necessarily determine the helical propensity of the sequence in the simulations. To minimise these effects, we used an optimised force field here [32]. With these caveats, we used the simulations of the five permutants to evaluate any changes in the structural properties monitored by the two types of spectroscopy used experimentally.

First, we consider the secondary structure content, monitored in experiments using CD. In our simulations, we find a consistent decrease in the helicity, relative to the WT simulations. For the WT, the average number of α-helical residues using a DSSP criterion [33] is 70, very close to the 73 helical residues of the experimental structure. For the mutants, the average number of residues in α-helical conformation is lower, as observed experimentally, within a range from 62 to 67 residues (Fig. S2). Second, we attempt to explain the results from fluorescence spectroscopy. Modelling tryptophan fluorescence from simulation results is extremely challenging, well beyond the scope of this work [34]. Instead, we can monitor the changes in the environment of the two Trp residues. In practise, we calculated the total accessible surface area of these amino acid residues [35] to assess whether permutation substantially modified their accessibility to solvent molecules (Fig. S3). Although, again, the simulations are too short to be absolutely conclusive, we find that the distribution of distances for the permutants consistently overlap with that of the WT, suggesting that the Trp residues remain similarly buried in the permutants. This is consistent with the relative insensitivity of the tryptophan fluorescence found in experiments.

Finally, we also monitored the global perturbations of the structure, which are relatively small: the largest RMSD from the initial structure occurs at the respective termini for each permutant and remains around a value of 2 Å for the α-helical segments (Fig. S4). Representative structures for each of the permutants were chosen from the most populated cluster observed in the MD simulations (Fig. S5). The contacts observed in the WT are maintained in the permutants. From all of these observations, we conclude that the simulations suggest only a small perturbation of the native structure due to the permutation and a modest decrease in the helicity, in agreement with the experiments.

### Effect of permutation on folding, stability and kinetics

**Fig. 2:**
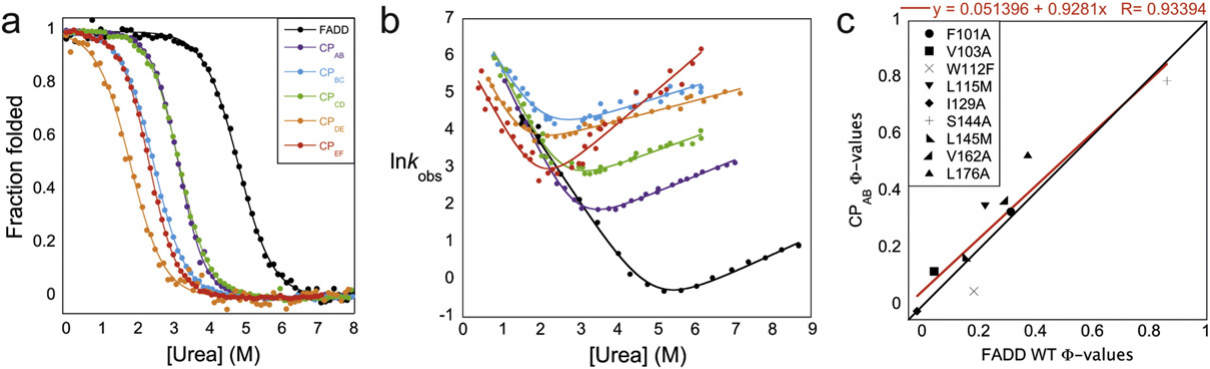
Thermodynamic and kinetic data for FADD WT and circular permutants. Constructs coloured as before. All experiments were carried out at 25 °C in 50 mM sodium phosphate (pH 7.0), 150 mM NaCl and 5 mM DTT and a final protein concentration of 1–2 μM. Data were analysed using Kaleidagraph (Synergy Software). (a) Fluorescence equilibrium curves. All permutants were destabilised compared to WT. Measurements were taken on a PerkinElmer fluorimeter with excitation at 280 nm and emission between 300 and 400 nm. Average emission wavelength was calculated in order to plot the data. (b) Chevron plots showing the dependence of the observed rate constant on urea concentration. Unfolding kinetics were monitored by changes in the fluorescence signal above 350 nm in a stopped-flow fluorimeter (SX20; Applied Photophysics) with 1:10 mixing. All kinetic traces were best described by a single-exponential equation as described previously [4]. (c) Comparison of Φ-values for WT FADD and CP_AB_. The relationship can be described by a straight line (red) with slope 0.9 ± 0.1 and an intercept close to 0, indicating that the Φ-values are essentially unchanged by permutation. Black line indicates a slope of 1.

Stability was determined by equilibrium urea denaturation experiments. All permutants demonstrated cooperative, reversible unfolding but had a range of stabilities and none was as stable as WT protein (Fig. 2a and Table 1). All *m-*values were similar to WT and well within the range found for previous studies of point mutations of FADD {see Ref. [4], 1.1–2.1 kcal mol^− 1^ M^− 1^ (mean, 1.41), standard deviation, 0.22}. The most destabilising circular permutant disrupted the long D–E loop that crosses and packs against the central hydrophobic core. Disruption of the other cross-core A–B loop is much less destabilising, likely because this loop makes fewer contacts.

**Fig. 3:**
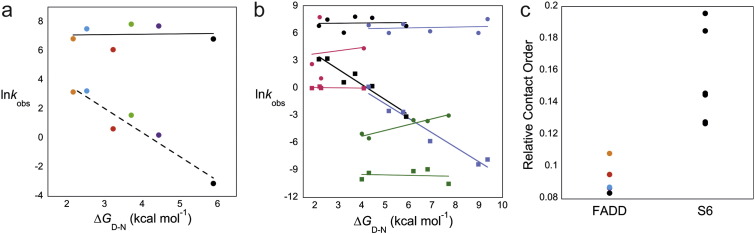
Plots describing the relationships between kinetics and stability. (a) ln*k*_u_ (broken line) and ln*k*_f_ (continuous line) plotted against ∆*G*_D-N_ for FADD WT and permutants, identified by colour as described previously. Results indicate that the refolding rate constant is unrelated to protein stability, but there is a strong correlation between ln*k*_u_ and ∆*G*_D-N_ (*R* = 0.96). Lines of best fit are shown. (b) Plot of ln*k*_u_ (squares) and ln*k*_f_ (circles) against ∆*G*_D-N_ for FADD (black), S6 [3] (blue), SH3 [1] (magenta) and IL-1β [23] (green). Lines of best fit are shown. (c) Plot showing the range of relative contact orders for permutants of FADD and S6 [3]. The relative contact orders as defined in Ref. [2] of FADD permutants were calculated from the MD structures.

WT FADD has been shown to have simple, monophasic folding and unfolding kinetics [4]. All circular permutants behaved in a manner consistent with this. Remarkably, despite the significant changes in connectivity, as well as in stability, the folding rates of the circular permutants were all very similar to WT (Fig. 2b and Table 1). The rate constants deviated from WT by only about a factor of 2. Importantly, since the folding limbs of the chevron plot almost overlay, and the denaturant dependence of the rate constants for folding (*k*_f_) are essentially the same as WT, we might infer that the rate-limiting transition state is similar to that of WT, at least in terms of relative collapse. As can be seen from the chevron plots, the loss of stability of the circular permutants of FADD is all reflected in the unfolding kinetics—all unfold significantly faster than WT (Figs. 2b and 3a). On reflection, this should not be a surprise since our previous results showed that the loops pack relatively late. Indeed, when we determine the Φ-value of the permutants, by treating them as mutants, we find that the Φ-values are all relatively low and similar to Φ-values of residues that are closest to each of the corresponding loops determined previously [4].

We note that the unfolding limb of one of the proteins, CP_EF_, is different to all other proteins; the slope of the unfolding limb (the unfolding *m*-value) is about double that of the other proteins, although the folding and equilibrium behaviour is essentially the same as the other proteins in the study. We cannot explain this behaviour at present. The obvious explanation is that the starting material, the folded protein, is a domain-swapped dimer under the conditions used in our studies (starting protein concentration before dilution, ~ 11 μM). In support of this suggestion, we note that this permutant leaves helix F somewhat detached from the rest of the protein, as it is joined only by the elongated new F–A loop introduced to join the original C- and N-termini—this would be likely to facilitate domain swapping at high protein concentrations. However, analytical size-exclusion chromatography (at 20 μM protein) did not detect any difference (within error) between CP_EF_ and all the other proteins (although, since FADD is an elongated molecule, it is possible that the cross-sectional area of a strand-swapped dimer is not different to an isolated domain) (Fig. S6). Moreover, unfolding experiments starting from a lower protein concentration (4.4 μM, the lowest protein concentration that allowed us to see a signal) gave unfolding rates over a range of denaturant concentrations that were indistinguishable. Despite this, the most likely explanation remains that CP_EF_ is actually a strand-swapped dimer—it is the protein that had a significantly altered Trp fluorescence profile (Fig. S1b). Note that we did not continue our studies of this circular permutant further.

### Permutation has no effect on folding mechanism

The central 4-helix motif in FADD comprises two pairs of parallel helices (A–E and B–D) packed together orthogonally. Thus, the early formation of the central core, which we observe to be the first step in the folding of WT protein, involves bringing together helices quite separated in sequence. Two of the permutants make the structure of FADD significantly simpler; CP_AB_ and CP_DE_ convert the protein into two associated 3-helix bundles. Thus, these were considered the two proteins most likely to have an altered folding mechanism. We hypothesised that each 3-helix bundle would now form early (as the contacts were more local) and then the two helical bundles would assemble late in the folding. Since CP_DE_ was significantly destabilised, we carried out a partial Φ-value analysis of CP_AB_ to determine whether the folding mechanism had, in fact, changed.

A selection of mutants for the Φ-value analysis was chosen from those performed by Steward *et al.* [4] to ensure that both the central core and the 3-helix bundles were probed. In each case, the same mutation was made in the permutant as in WT FADD, with the exception of Trp112, which was mutated to Phe rather than Ala to avoid extensive destabilisation. All mutant chevrons fit well to the linear fit used for CP_AB_; Φ-values were calculated from refolding data at 2 M urea for consistency with the WT Φ-value analysis (to reduce error from extrapolation to 0 M denaturant). The pattern of Φ-values was the same as that for WT, and even the absolute values of Φ had not altered significantly (Fig. 2c and Table S1): the folding mechanism is remarkably unchanged.

### Comparison with other studies

We compared our kinetic results with other studies that had investigated the folding kinetics of three or more permutants; SH3, IL-1β and S6 [1,3,23]. All of these are predominantly β structures: SH3 is an all-β barrel-like structure, IL-1β has a β-trefoil fold but S6 is a Greek key β-sandwich and is thus structurally similar (albeit primarily β) to FADD with two loops that cross-over the core at either end of the domain (1U06, 1I1B and 1RIS [36–38]). Linear free energy relationship plots (Fig. 3a and b) show that our observation, that folding rate constants are relatively insensitive to permutation, is neither unique nor the norm. The two Greek key proteins, FADD and S6, behave in an extremely similar manner: circular permutation alters stability and unfolding kinetics but has remarkably little effect on the rate of folding. This is not the case for either SH3 or IL-1β, where, if anything, the effect is on folding kinetics. This suggests that, in these Greek key proteins (as has been seen previously [39–41]), the loop regions play little role in nucleating folding—the same may not be true for SH3 and β-trefoil proteins (although there are too few examples to be certain).

In other respects, FADD and S6 behave quite differently. In their studies of S6, Lindberg *et al*. observed that permutation was accompanied by a radical shift in the folding mechanism, with the site of nucleation shifting in the circular permutants [20]. Our data suggest that this is not the case for FADD. How can this be explained? The difference between S6 and FADD is that the first is all-β and the other is all-α. Contact order is a measure of the average separation in sequence between residues that are in contact with each other [2]. If we examine the contact order of S6 and FADD, we see that they are very different, as one would expect from an all-β and an all-α protein [42]. Importantly, however, in the all-β S6, permutation alters the relative contact order significantly, in particular, it alters the relative separation of residues that nucleate folding (described as ∆L for the S6 system [43]); thus, the entropic cost of forming alternative nuclei can be radically altered by permutation. In all-α FADD, on the other hand, we observed very little change in contact separation since local helical contacts are so dominant that permutation has little effect on the contact order (Fig. 3c). Thus, we infer that the entropy of the folding nucleus is insensitive to permutation; thus, no alternative, lower-entropy cost nuclei are favoured by permutation and the folding mechanism remains the same. Plasticity of the folding nucleus may be of greater importance in β-sheet proteins with critical long-range contacts than in α-helical proteins where nucleation of folding requires formation of mostly local interactions; this may explain why the Greek key motif is common amongst β-sheet proteins but only found in a minority of related all-α proteins [44]. Our results suggest that the type of secondary structure is the determining factor in the balance between topology and connectivity.

## Figures and Tables

**Table 1:** Thermodynamic and kinetic parameters for FADD WT and circular permutants.

FADD construct	New N-terminus	$\Delta \Delta G_{D‐N}^{H_{2}O}$ (kcal mol^− 1^)^a^	*m*_D-N_ (kcal mol^− 1^ M^− 1^)	$k_{f}^{H_{2}O}$ (s^− 1^)	$k_{u}^{H_{2}O}$ (s^− 1^)	Φ^b^
WT	—	—	1.4 ± 0.2	940 ± 110	0.04 ± 0.01	—
CP_AB_	K110	1.74 ± 0.16	1.59 ± 0.02	2300 ± 200	1.2 ± 0.1	0.11
CP_BC_	V121	3.43 ± 0.15	1.34 ± 0.01	950 ± 170	24.3 ± 1.7	− 0.01
CP_CD_	R135	2.24 ± 0.15	1.42 ± 0.01	1900 ± 400	26.1 ± 4.4	0.05
CP_DE_	E154	4.44 ± 0.16	1.27 ± 0.03	2600 ± 500	4.9 ± 0.9	− 0.01
CP_EF_	M170	2.77 ± 0.15	1.66 ± 0.01	450 ± 80	1.9 ± 0.5	0.32

The errors quoted are the errors of the fits of the data.

a The change in free energy of unfolding was determined from analysis of the equilibrium denaturation data (Fig. 2a) as follows: ∆∆*G*_D-N_ = ∆*G*_D-N_^WT^ − ∆*G*_D-N_^CP^, where ∆*G*_D-N_ = *m*[urea]_50%_ ([urea]_50%_ is the midpoint of the transition).

b Calculated from the rate constants for folding (*k*_f_) at 2 M urea (see the text) as follows: Φ = ∆∆*G*_D-TS_/∆∆*G*_D-N_, where ∆∆*G*_D-TS_ = *RT*ln(*k*_f_^WT^/*k*_f_^CP^). Error in Φ is generally considered to be < 0.1.

## Appendix A Supplementary data

This contains Supplementary Figure S1 - S6, and Supplementary Table S1, with associated references.
