# Supplementary material for: The Response of Greek Key Proteins to Changes in Connectivity Depends on the Nature of Their Secondary Structure
Source: J Mol Biol. 2015 Jun 19;427(12):2159–65. doi: 10.1016/j.jmb.2015.03.020 (PMC4451459; doi:10.1016/j.jmb.2015.03.020)
Supplement: Supplementary file 1 — This contains Supplementary Figure S1 - S6, and Supplementary Table S1, with associated references. [file mmc1.pdf]

Supplementary information

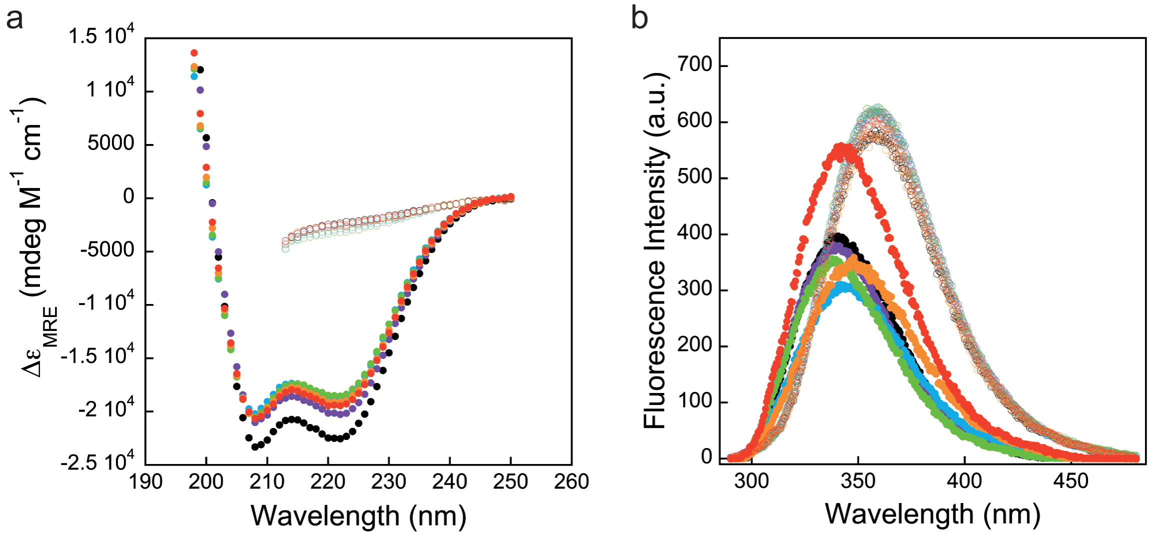

Figure S1: CD and fluorescence spectra for FADD wild-type and circular permutants. Unfolded proteins are shown as open circles, folded protein as closed circles; colour-coded as in main text: **black**, WT; **purple**, CP_{AB}; **blue**, CP_{BC}; **green**, CP_{CD}; **orange**, CP_{DE} and **red**, CP_{EF}. (a) CD spectra for FADD and permutants. Measurements were taken using a Chirascan CD spectrometer (AppliedPhotophysics). Data were analysed in Kaleidagraph (Synergy Software), between the permutants there is little difference in the signal at 222 nm, suggesting that the secondary structure content is relatively consistent. Note that the FADD wild-type is 3 residues shorter than the permutants. (b) Fluorescence scans of FADD and permutants. Measurements were taken using a LS-55 luminescence spectrophotometer (PerkinElmer) and data were analysed in Kaleidagraph. Possible reasons for shifts in the fluorescent behaviour of the native-states are discussed in the main text.

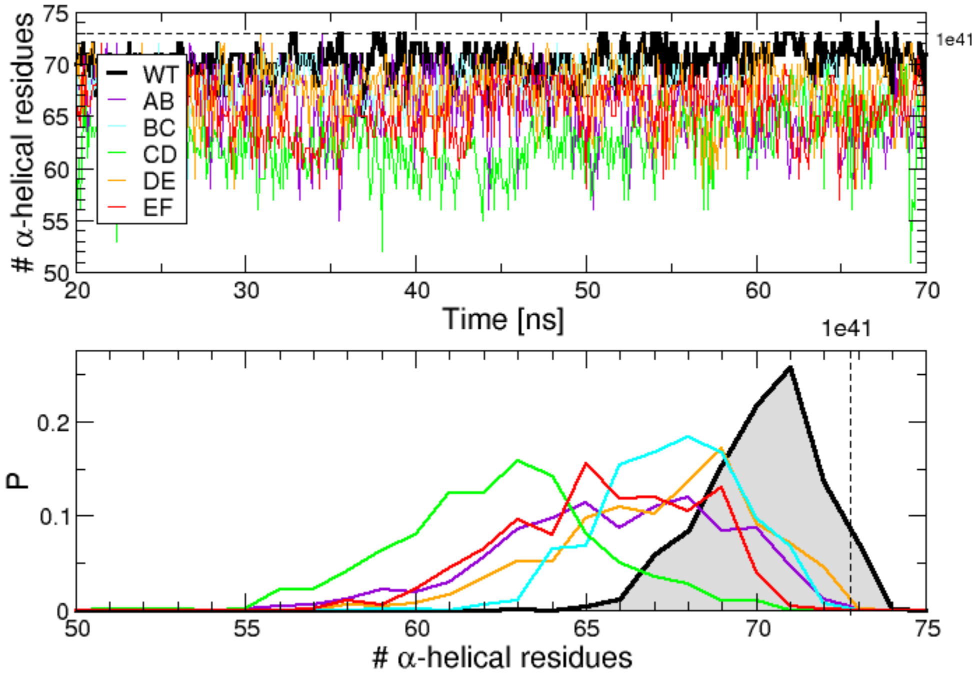

Figure S2: Helicity of FADD and permutants by simulation. Top: Time series for the number of alpha helical residues of the WT FADD and permutants according to the DSSP criterion in the productive part of the MD simulation runs (20-70 ns). Bottom: Population of the number of helical residues. In both panels, the value corresponding to the WT structure is shown as a dashed line.

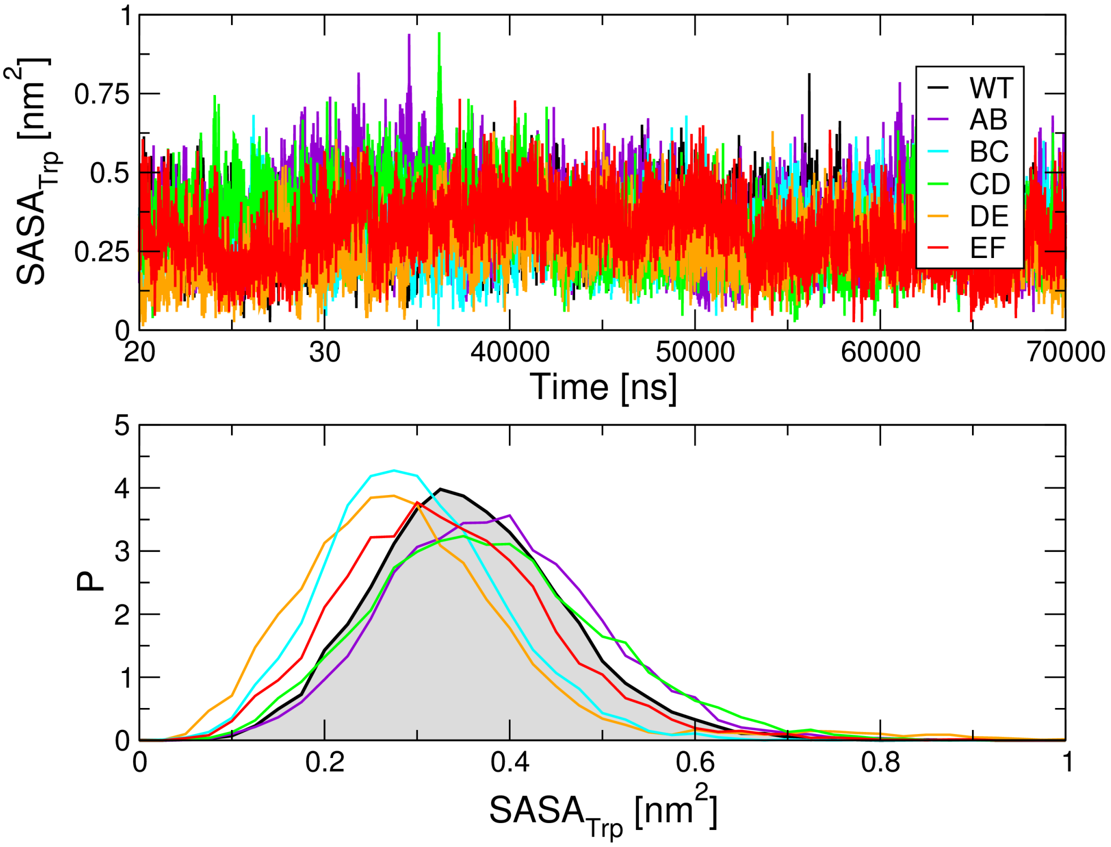

Figure S3: Effect of circular permutation of Trp exposure. Top: Time series for the solvent accessible surface area of the tryptophans ($SASA_{Trp}$) in the WT and permutants of the death domain. Bottom: Population of the $SASA_{Trp}$.

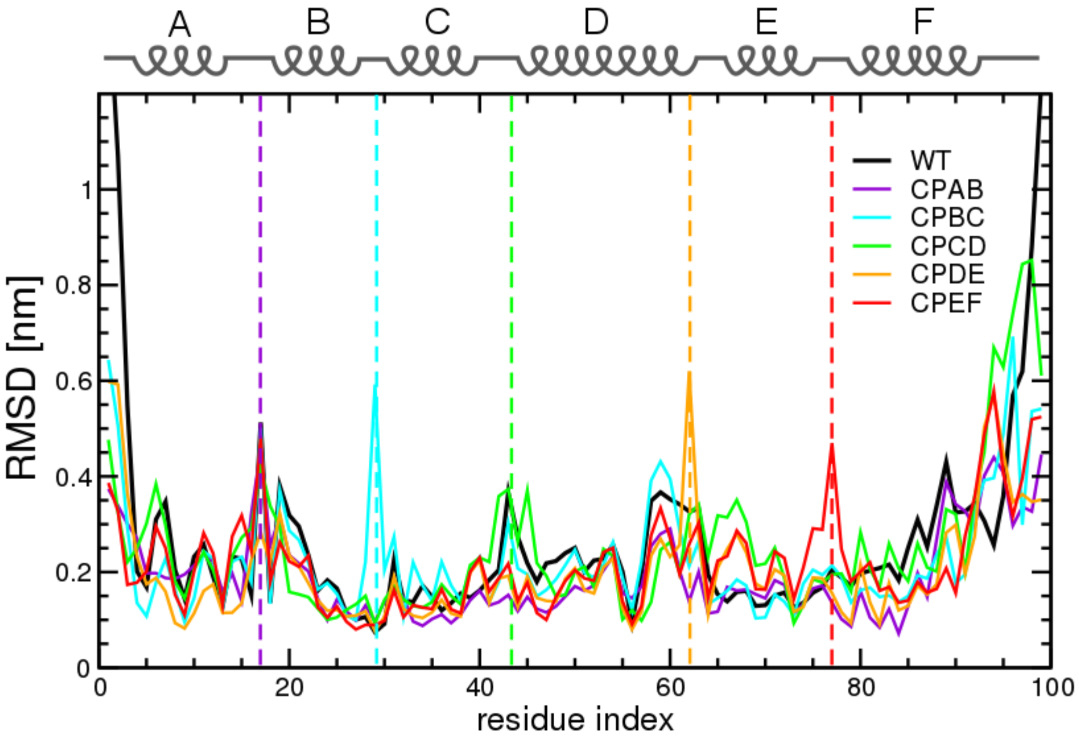

Figure S4: Residue-wise RMSD in the atomistic simulations of WT FADD and five permutants of the death domain. All sequences are overlaid on the WT, and the new termini for each sequence are indicated with a dashed line. The cartoon at the top shows the regions corresponding to the helical segments.

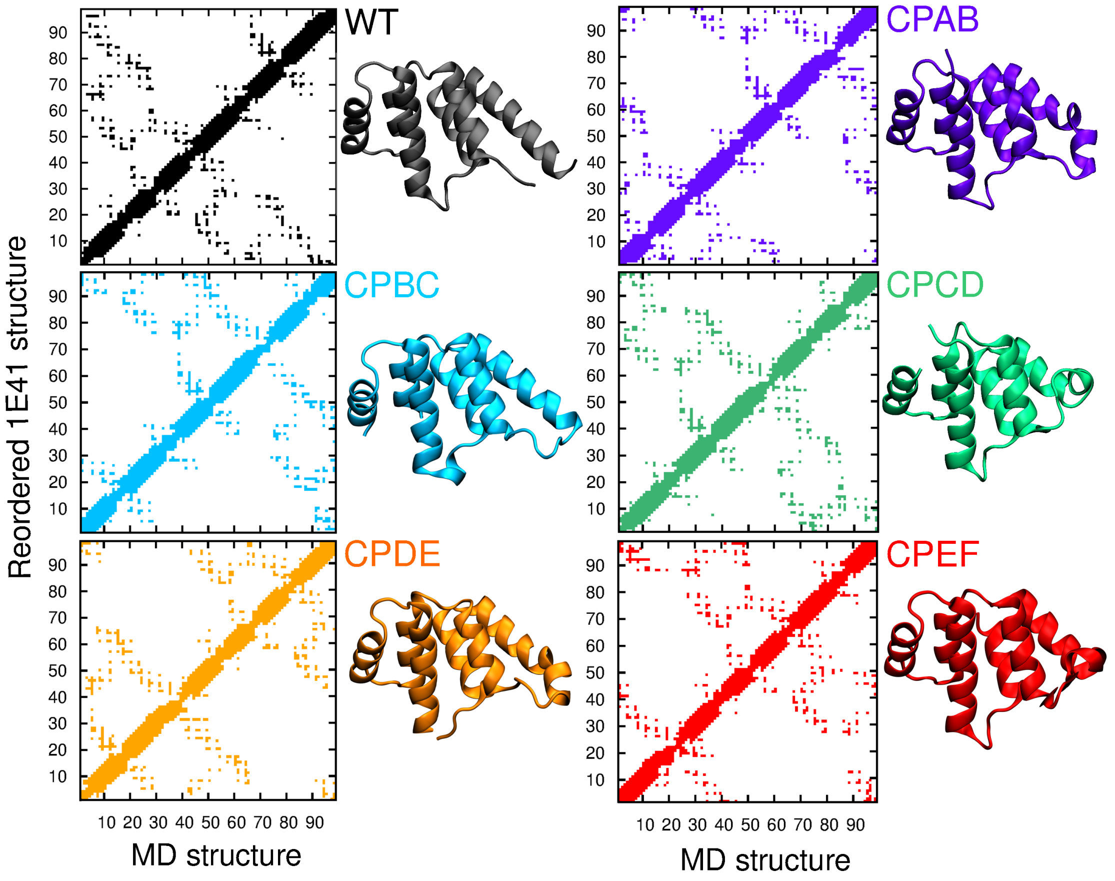

Figure S5: Contact maps and simulated structures of FADD and its permutants (colours as throughout). For each of the permutants considered in this study, initial structures were generated manually using the experimental NMR models from the WT death domain (PDB id: 1e41[1]). For the permutants, the experimental sequence was produced, excluding the GSGSS N-terminal tails. For both the WT and the permutants, the same simulation methods were used. The Amber03* force field [2] was used for the protein. First, we solvated the protein in an octahedral simulation box of TIP3P water molecules [3] of 6 nm length in the longest dimension. Then, the protein and water coordinates were energy minimized using a steepest descent algorithm. Using the minimized coordinates, a 200 ps simulation with position restraints was run in the NVT ensemble, using a stochastic thermostat. The result of this simulation was used to initialize a 50 ns MD simulation. Newtonian dynamics were propagated using a leap-frog algorithm, with a 2 fs time-step. In all cases the particle-mesh Ewald method was used for the long-range electrostatics [4]. Pressure was kept at 1 Pa with the Parrinello-Rahman barostat [5] and temperature was kept at 300 K using the Berendsen thermostat with a coupling constant of 0.1 ps [6]. All simulations were carried out using the Gromacs simulation package (version 4.5) [7]. For each system we clustered the coordinates of the equilibrium run using the Daura algorithm with a 1 Angstrom cut-off, [8] and chose the most populated cluster for estimating the contact map and the contact order. The corresponding contact maps are shown next to the structure of each construct. The bottom right section of each plot shows the contact map calculated from the MD simulated structures. The NMR structure [1E41] was used to calculate a contact map for the wild-type, and was

reshuffled to construct maps for the permutants as well, these are shown in the top left. Essentially, the circular permutants of FADD retain the native interactions.

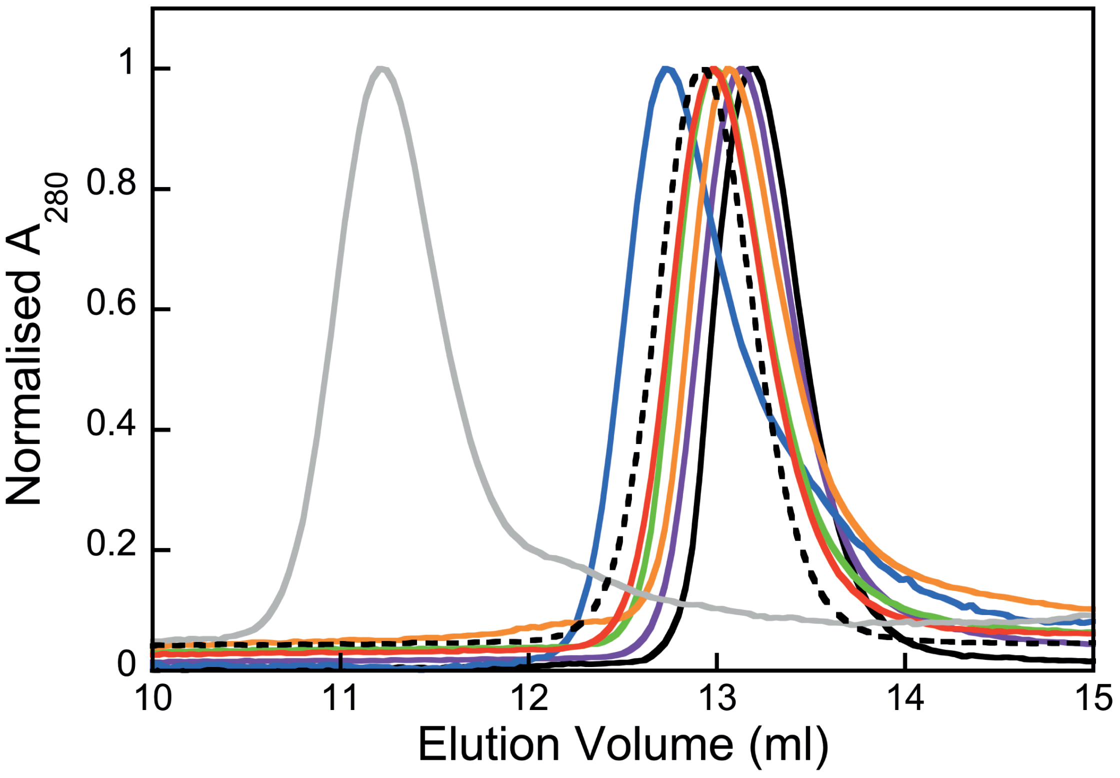

Figure S6: Analytical size exclusion chromatograms. WT FADD, circular permutants (coloured as in main text) and a covalently attached dimer of WT (**grey**) were run down a G75 10/300 column (GE Healthcare) at 0.5 mL/min. 200 µL of each sample was loaded onto the column at a protein concentration of 20 µM in buffer (see Figure 2). The dashed and solid **black** lines represent 2 runs of wild-type, the difference in these runs suggests that shifts seen in other peaks may not indicate structural changes. Note that WT FADD is 3 residues shorter than the permutants.

Table S1: Thermodynamic and kinetic parameters for CP_{AB} phi-value mutants.

CP_{AB} variant	Helix/Core probed by mutation	$\Delta\Delta G_{D-N}^{H_2O}$ (kcal mol^{-1})^{a}	k_f^{2M} (s^{-1})	Φ^{b}
WT	-	-	35 ± 3.0	-
F101A	A Central	3.3 ± 0.05	6 ± 0.3	0.33
V103A	A Bundle 1	0.7 ± 0.05	31 ± 2.7	0.12
W112F	B Bundle 2	1.7 ± 0.05	33 ± 1.1	0.05
L115M	B Central	1.3 ± 0.05	24 ± 1.4	0.35
I129A	C Bundle 2	2.4 ± 0.05	38 ± 2.6	-0.02
S144A	D Bundle 2	-0.7 ± 0.05	93 ± 4.9	0.79
L145M	D Central	1.6 ± 0.06	14 ± 0.6	0.17
V162A	E Bundle 1	3.2 ± 0.05	7 ± 0.3	0.37
L176A	F Bundle 1	1.1 ± 0.05	13 ± 0.4	0.53

a The change in free energy of unfolding was determined from analysis of the equilibrium denaturation data as described for Table 1.

b Φ was calculated from the rate constants for folding (k_f) at 2M urea (see text) as for Table 1. Error in Φ is generally considered to be < 0.1.

References

[1] Berglund H, Olerenshaw D, Sankar A, Federwisch M, McDonald NQ, Driscoll PC. The three-dimensional solution structure and dynamic properties of the human FADD death domain. J Mol Biol. 2000;302:171-88.

[2] Best RB, Hummer G. Optimized molecular dynamics force fields applied to the helix−coil transition of polypeptides. J Phys Chem B. 2009;113:9004-15.

[3] Jorgensen WL, Chandrasekhar J, Madura JD, Impey RW, Klein ML. Comparison of simple potential functions for simulating liquid water. J Chem Phys. 1983;79:926-35.

[4] Darden T, York D, Pedersen L. Particle mesh Ewald: An N·log(N) method for Ewald sums in large systems. J Chem Phys. 1993;98:10089-92.

[5] Parrinello M, Rahman A. Polymorphic transitions in single crystals: A new molecular dynamics method. J Appl Phys. 1981;52:7182-90.

[6] Berendsen HJC, Postma JPM, van Gunsteren WF, DiNola A, Haak JR. Molecular dynamics with coupling to an external bath. J Chem Phys. 1984;81:3684-90.

[7] Pronk S, Páll S, Schulz R, Larsson P, Bjelkmar P, Apostolov R, et al. GROMACS 4.5: a high-throughput and highly parallel open source molecular simulation toolkit. Bioinformatics. 2013;29:845-54.

[8] Daura X, Gademann K, Jaun B, Seebach D, van Gunsteren WF, Mark AE. Peptide Folding: When Simulation Meets Experiment. Angew Chem Int Edit. 1999;38:236-40.
